# A drift–diffusion decomposition of conditions that influence shallow (“good enough”) processing of heard sentences

**DOI:** 10.3758/s13421-025-01748-3

**Published:** 2025-06-23

**Authors:** Ryan M. O’Leary, Natalie Omori-Hoffe, Griffin Dugan, Arthur Wingfield

**Affiliations:** 1 Department of Psychology and Volen National Center for Complex Systems, Brandeis University, Waltham, MA, USA

**Keywords:** Speech perception, Language comprehension, Syntactic processing, Speed-accuracy trade-off, Drift–diffusion models

## Abstract

Although listeners may have the competence to engage in a word-by-word analysis to build a syntactic representation of a heard sentence, in everyday conversation, listeners may, almost by necessity, process the sentence only to a shallow or “good enough” level to derive its meaning. The possibility has been raised that processing schemata may be flexible, such that under some circumstances, comprehension decisions are more likely to be based on an incomplete analysis. We report two experiments in which adult participants were tested on their ability to determine the meaning of auditorily presented plausible or implausible sentences. In both experiments, participants were given two different orienting instructions while completing the listening task (instructions that emphasized either the speed or accuracy of the comprehension decision). In Experiment 1, we also manipulated spectral clarity such that speech was either heard clearly or degraded in spectral richness. A unique use of hierarchical drift–diffusion modeling was employed to probe latent decision-making processes that influenced the participants’ comprehension decision. Results indicate that orienting instructions that emphasize speed and perceptual challenge both increase the likelihood that the assumed meaning of implausible sentences will be based on plausibility. Drift–diffusion modeling revealed a dissociation where orienting instructions selectively influenced the amount of evidence required for the participant to make a comprehension decision while sentence plausibility selectively influenced the rate of evidence accumulation. These drift–diffusion model findings were replicated in Experiment 2. Results suggest that comprehension processes are highly flexible and can be characterized in terms of underlying decision-making mechanisms.

## Introduction

Some 70 years ago in his seminal monograph, *Syntactic Structures,* Chomsky emphasized the distinction between linguistic competence and everyday language use. He further argued that the former should be the target for the development of a theory of syntax (Chomsky, 1957). As theories of sentence comprehension evolved over the years the emphasis on competence was generally maintained as a platonic ideal of sentence processing in which the listener matches the acoustic input against entries in their mental lexicon (cf., Luce et al., 1998; Marslen-Wilson & Zwitserlood, 1989; McClelland & Elman, 1986) and develops a hierarchical representation of the syntactic structure of the sentence, thus allowing access to the sentence meaning (Chomsky & Miller, 1963; Fodor & Bever, 1965; MacDonald et al., 1994).

In the everyday world, however, much of what we hear in daily conversation consists of sentence fragments, ungrammatical utterances, or sentences with underspecified syntax (Elsness, 1984; Goldman-Eisler, 1968; Thompson & Mulac, 1991), with the listener’s understanding of the spoken content supported by context-based inference combined with an assumption that the meaning conveyed is plausible (Gibson et al., 2013; Hale, 2001; Padó et al., 2009). Although our focus here is on the understanding of spoken sentences, this principle can also appear with written text. For example, seeing a sign in a hospital emergency room that says “No head injury is too trivial to be ignored” leaves no doubt in the viewer’s mind that the implication of the sign is to take even a minor head injury seriously. It is a rare observer who notices that the sign was poorly phrased and in fact literally means just the opposite (Wason & Reich, 1979). The point of this example of a so-called ‘depth-charge illusion’ sentence is that, as often as not, we weigh plausibility over the literal meaning of a sentence (Zhang et al., 2023).

In hearing spoken sentences the process of sampling a few key (typically stressed) words and using plausibility to determine the meaning of a sentence has been referred to as “good enough” or “shallow” processing (Christianson, 2016; Christianson et al., 2001; Ferreira & Patson, 2007; Ferreira et al., 2001; Frances, 2024; Sanford & Sturt, 2002). That is, although listeners may have the competence to engage in a word-by-word analysis to build a syntactic representation of a sentence, in everyday conversation, with speech arriving at average rates of 140–180 words per minute, listeners may, almost by necessity, process the sentence only to a shallow or “good enough” level to determine its meaning.

When a plausible sentence is heard, however, one cannot easily tell whether the listener has conducted a full lexicosyntactic analysis or has engaged in shallow processing, since both algorithms would yield the correct meaning. In the case of syntactically simple sentences heard under ideal listening conditions, a full syntactic analysis of a heard sentence may precede extraction of a sentence meaning. It has been demonstrated, however, that when sentences have a more complex structure, listeners may often engage in shallow processing (e.g., Amichetti et al., 2016; Ayasse et al., 2021). This distinction between meaning based on a full syntactic analysis versus shallow processing has been demonstrated by testing comprehension of implausible syntactically complex sentences that, if fully analyzed, would reveal that the sentence in fact expresses an unlikely or implausible meaning. Whether such a sentence had received shallow processing would be revealed by the listener giving a plausible, if incorrect, interpretation (e.g., Amichetti et al., 2016; Ferreira et al., 2002; Ferreira, 2003). If a listener does indeed conduct a word-by-word analysis constructing a syntactic representation of the sentence, its literal meaning, and hence its implausibility, would be revealed. Because we live in a plausible world, and most sentences we hear have a plausible meaning, shallow processing would typically yield the correct meaning and do so with less cognitive effort than conducting a full syntactic analysis (Ayasse et al., 2021).

A well-known case of shallow or “good enough” processing occurs when sentences contain a noncanonical order of thematic roles, such as object-relative clauses. There are several accounts for why these sentences are more demanding than sentences with subject-relative clauses. One account is that these noncanonical sentences violate expectations about thematic role assignment, as the first noun in the sentence is not the agent of the action. This violation of expected placement in a sentence may require more extensive integration to interpret correctly (Warren & Gibson, 2002). Noncanonical sentences are also relatively infrequent in everyday communication, which may further contribute to processing difficulty by increasing the likelihood of misinterpretation or the need for reanalysis (Gibson et al., 2013; Goldman-Eisler, 1968; Levy, 2008; Padó et al., 2009). It has also been suggested that the difficulty derives from the greater demands object-relative sentences place on working memory as compared to subject-relative sentences (Cooke et al., 2002). For whatever reason, it is well established that comprehension errors are more likely when sentences have object-relative clauses as compared to subject-relative clauses (Cooke et al., 2002; Just & Carpenter, 1992). Importantly, when an object-relative or another noncanonical sentence is paired with an implausible assignment of thematic roles, listeners often reverse the thematic roles in order to derive a plausible yet syntactically incorrect interpretation (Christianson et al., 2010; Christianson et al., 2023; Ferreira, 2003; Lim & Christianson, 2013; Zhou & Christianson, 2016a, 2016b).

It has been suggested that, given the same sentence, there are circumstances that may make shallow processing more likely. Although not exhaustive, two such conditions are when a listener is placed under time pressure to make a decision as to a sentence meaning, or when comprehension must take place concurrently with operations that also require a draw on resources (Ferreira & Patson, 2007). Such expectations are in accord with Rönnberg and colleagues’ (2013) suggestion that processing schemata may be flexible, such that under some circumstances, comprehension decisions are based on either a detailed or an incomplete analysis.

**Fig. 1:**
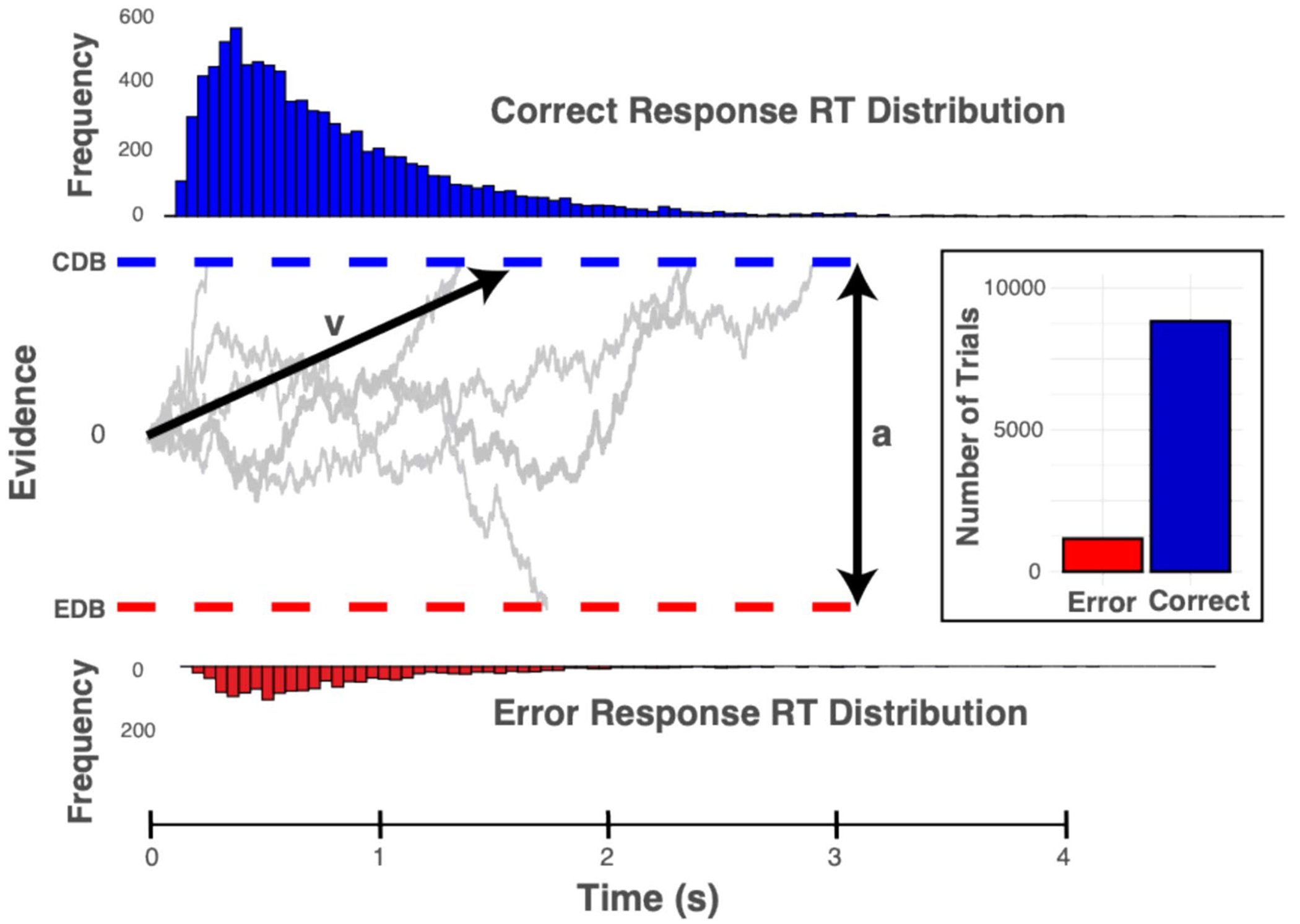
Visualization of a simplified drift–diffusion model (DDM) with simulated accuracy and response latency data. Evidence is noisily accumulated over time until the threshold is hit for a correct decision (correct decision boundary; CDB) or an error (error decision boundary; EDB). Key parameters of interest for the present study are shown as v; the drift rate, which accounts for the rate of evidence accumulation towards the correct decision boundary and a; decision boundary separation, which accounts for the amount of evidence required for a choice to be made. Corresponding response latency distributions for correct responses are shown above the correct decision boundary, while response latency distributions for error responses are shown below the error response decision boundary

In the present study, we build on this notion by adopting a mechanistic account of sentence comprehension that characterizes the decision-making processes that underlie comprehension responses under challenging conditions. To investigate latent decision-making parameters, we employed hierarchical Bayesian drift–diffusion models (HDDMs; Wiecki, et al., 2013). HDDMs are a multilevel Bayesian extension of the well-established drift–diffusion model (DDM; Ratcliff & Rouder, 1998). We have chosen DDMs because of their ability to model speed-accuracy tradeoffs in two-choice tasks within a single unified model (see Ratcliff et al., 2016, for a review). These models estimate interpretable latent decision-making parameters using the response accuracy and response latency distributions that are not apparent in the behavioral data. A visualization of simulations from a simplified drift–diffusion model is shown in Fig. 1.

The first component we wished to investigate is how challenges to sentence comprehension impact the threshold that must be reached for a decision to be executed (referred to below as the decision boundary separation). The second decision-making component of interest is the rate of accumulation of evidence toward the correct response (referred to as the drift rate), and how that rate may vary based on the experimental condition.

The DDM approach assumes decision-making is a process of accumulating evidence amidst noise until a decision threshold is reached. Key parameters of the DDM include the drift rate (*v*), which represents the average rate of evidence accumulation; the decision threshold (*a*; decision boundary parameter, often referred to as response caution), which determines the amount of evidence required for the choice to be made; the starting point, which indicates potential biases toward a choice; and the non-decision time, which adjusts for other task facets such as motor response. While these parameters unfold over the same response interval, they index different decision-making dynamics. For example, a high decision boundary indicates that participants respond cautiously (slowly but accurately), while a high drift rate indicates that participants are responding quickly and accurately. Conversely, a low decision boundary parameter results in quick but error-prone responses, while a low drift rate results in slow and error-prone responses. A low drift rate is suggestive of greater difficulty or uncertainty in interpreting the stimulus, regardless of how cautious the decision strategy is. A higher decision boundary suggests a more cautious decision style where more information is required before a response is executed.

Experiment 1 served as a proof-of-concept exploration of two real-world circumstances that may increase the likelihood of shallow or ‘good enough’ sentence processing and examined the extent to which these factors influence latent decision-making mechanisms. Experiment 2 was conducted after the completion of Experiment 1 in order to replicate the major drift–diffusion model findings. In both experiments, object-relative clause sentences would be heard that contained an action, the agent of the action, and the recipient of the action. In the majority of cases the sentences would be plausible but in some cases the sentence meaning would be implausible. In either case the sentence would be followed by a statement naming the agent or the recipient of the action, which might be true or false. Focusing on the implausible sentences, our goal was to compare the likelihood that the listener evidences an understanding of the literal, if implausible, meaning, as opposed to selecting the plausible, although incorrect, meaning.

## Experiment 1

Experiment 1 examined two experimental manipulations and their influence on the processing of plausible and implausible sentences, as well as the joint effects of these factors on latent decision-making parameters. In one condition we examined the effect of time pressure, with the hypothesis that giving orienting instructions to respond rapidly would yield more instances of shallow processing than orienting instructions that emphasized accuracy. In the second condition we tested effects of listening conditions, in which we used noise-band vocoding to simulate the limited spectral richness of speech heard via a cochlear implant (Cychosz, et al., 2024; Shannon et al., 1995). We selected a simulation of cochlear implant listening both because of the increasing use of cochlear implants among adults whose hearing loss has developed beyond the benefit of conventional hearing aids (Lin et al., 2012), and because their lack of spectral richness along with other sources of distortion (Friesen et al., 2001; Fu & Nogaki, 2005; Perreau et al., 2010) make speech comprehension especially effortful (e.g., O’Leary et al., 2023; Winn et al., 2015). We hypothesized that hearing a sentence that has been spectrally degraded by vocoding will be more likely to result in the listener using shallow processing than sentences heard with clear speech. In both the case of orienting instructions and acoustic manipulation we also measured response latencies to the participants’ comprehension responses.

To the extent that these hypotheses are sustained, however, one cannot say whether the same mechanism(s) underlies both effects. For this purpose, we applied DDMs as previously described. We hypothesized that when participants are instructed to prioritize speed relative to accuracy, that the amount of evidence required to reach a decision threshold (the decision boundary) will be reduced. While this finding has not, to our knowledge, been demonstrated in a sentence comprehension task, this hypothesis is based on several extant studies that found significant effects of task instructions on decision boundaries in perceptual discrimination-based decision-making tasks (Ratcliff, 2002; Ratcliff & Rouder, 1998; Ratcliff et al., 2003; Thapar et al., 2003) as well as in value-choice decision making tasks (Milosavljevic et al., 2010; Mormann et al., 2010). This result would indicate that listeners are able to flexibly adjust their response strategy to accommodate the change in the task instructions.

We further hypothesized that listening to sentences that are implausible or spectrally degraded will reduce the rate of evidence accumulation toward the correct decision relative to plausible or acoustically clear sentences, as evidenced by decreases in the drift rate parameter. Regarding vocoded speech, because the input is spectrally limited, there is less acoustic evidence available for the listener to accumulate from the degraded signal. Additionally, we expect a similar effect of sentence plausibility on drift rate. This effect may partially reflect the ease of semantic integration in plausible sentences. In contrast, a decrease in drift rate for implausible sentences may be explained by increased cognitive load (Ayasse et al., 2021), greater uncertainty, and the potential need for reanalysis.

### Method

#### Participants

Participants were 32 university students and staff (11 males and 21 females), with ages ranging from 18 to 25 years (*M* = 19.78 years, *SD* = 1.68). Participants received audiometric screening to ensure normal hearing. The audiometric evaluation was carried out using a Grason-Stadler AudioStar Pro clinical audiometer (Grason-Stadler, Inc., Madison, WI) using standard audiometric procedures in a sound-attenuating testing room. All participants had a better-ear pure tone threshold average (PTA) across 0.5, 1, 2, and 4 kHz of less than 25 dB HL, placing them in a range considered to be clinically normal hearing for speech (Katz, 2002).

All participants reported themselves to be native speakers of American English with no known medical conditions that might interfere with the ability to perform the experimental tasks. Written informed consent was obtained from all participants according to a protocol approved by the Brandeis University Institutional Review Board (IRB).

#### Stimuli

Preparation of the test stimuli began with the construction of 96 semantically plausible 8- to 11-word English sentences with an object-relative center-embedded clause structure (e.g., “The rabbit that the eagle attacked was large”). An implausible version of each sentence was then created by switching the agent of the action and the recipient of the action to produce a sentence with the same words and syntactic structure but representing an unlikely scenario (e.g., “The eagle that the rabbit attacked was large”). This yielded a master list of 192 test sentences (96 plausible and 96 implausible).

To discourage listeners from developing incidental processing strategies based on the uniform syntactic structure of the test sentences, an additional 192 filler sentences were created. The fillers consisted of 96 sentences with a subject-relative center-embedded clause structure (e.g., “The parent that scolded the toddler was tired”) and 96 sentences with an active declarative structure (“The dog chased the cat into the neighbor’s yard”). All of the fillers contained an action and an agent of the action, and all were semantically plausible. Performance on the filler sentences was not included in the results.

Creation of the test sentences and fillers was aided by ChatGPT (OpenAI, 2024) based on the criteria of sentence length, syntactic structure, and plausibility. Initial prompts included the stimulus set used by Amichetti et al. (2016) as examples of plausible sentences which, when their thematic roles were reversed, became implausible. All of the generated sentences were reviewed in terms of the specified criteria and edited, if necessary. Three independent researchers reviewed the sentences to confirm that they were indeed plausible, and that reversing the thematic roles created implausible sentences. Both test and filler sentences were spoken in a female voice in standard American English generated via an AI program (ElevenLabs AI; https://elevenlabs.io). The resultant recordings were equalized within and across sentences for root-mean-square (RMS) intensity using Praat (Boersma & Weenink, 2022).

##### Vocoding

Each of the 384 sentences (96 plausible test sentences, 96 implausible test sentences, 192 filler sentences) was processed to produce versions with six vocoder channels. Noise-band vocoding was conducted using MATLAB (MathWorks, Natick, MA) following the method described by Shannon et al. (1995; see also Kong et al., 2015). The specific parameters used in this vocoder simulation were a frequency range of 141–7,000 Hz, a 6 dB/octave roll-off pre-emphasis high-pass filter using a first-order Butterworth filter, and an 18 dB/octave roll-off third order Butterworth band-pass filter into logarithmically spaced frequency bands. Envelope extraction was conducted using a 36 dB/octave roll-off sixth order Butterworth low-pass filter at 300 Hz, and the low-pass filter cut-off of the amplitude envelope was 300 Hz. The degree of spectral impoverishment represented by six-channel vocoding was chosen to approximate speech quality associated with cochlear implant users who often receive speech with the equivalent of four to eight frequency channels (Friesen et al., 2001; Fu & Nogaki, 2005; Perreau et al., 2010; see also Faulkner et al., 2001).

#### Procedure

Participants were told that they would hear a series of recorded sentences, some of which would be in clear speech, and some would be degraded by a computer. After each sentence, the participants were presented with a sentence displayed on a computer screen that could be either true or false (e.g., “The eagle attacked the rabbit”). The participant’s task was to indicate whether the probe statement was true or false by pressing an indicated key on the computer keyboard. The probe sentences were counterbalanced across conditions such that the correct answer was equally likely to be “true” or “false.”

There were two orienting conditions. In an *accuracy condition,* participants were told that their goal was to be as accurate as possible in their decision responses and to strive for 100% accuracy. In the *speed condition,* participants were told their goal was rapid decision making. They were told that when the true/false question was presented they should strive to give their decision response as rapidly as possible. The presentation computer recorded the accuracy of participants’ responses in each of the two orienting conditions. No mention was made of the fact that the computer was also recording the time from when the question was presented to the keypress indicating the participant’s true or false response.

Participants heard a total of 192 sentences, 96 of which were test sentences. Of these, 48 were plausible and 48 implausible, with 24 of each heard with clear speech and 24 heard with six-channel vocoding. In turn, 12 of each were presented with accuracy instructions and 12 with speed instructions. The remaining 96 sentences were fillers, 48 heard with clear speech and 48 heard with six-channel vocoding, with 24 of each presented with accuracy instructions and 24 with speed instructions. As previously noted, all of the filler sentences were semantically plausible such that, overall, 75% of the sentences heard by the participants were semantically plausible.

Orienting conditions (accuracy vs. speed) were blocked in presentation, with half of the participants receiving the accuracy condition first and the remaining participants receiving the speed condition first. Within each orienting condition sentence type (true/false, clear/vocoded, test/filler) were intermixed in presentation. The particular test sentences heard in each condition were counterbalanced across participants, such that, by the end of the experiment, each test sentence had been heard an equal number of times in each condition.

Sentences were presented binaurally over Eartone 3 A insert earphones (E-A-R Auditory Systems, Aero Company, Indianapolis, IN, USA) at 65 dB HL. Prior to the main experiment, audibility was affirmed by presenting ten two-syllable words, one at a time, at 65 dB HL with instructions to repeat each word as it was presented. All participants achieved 100% accuracy. Following this, participants underwent a vocoded speech adaptation phase to familiarize the participants with the sound of vocoded speech. During the adaptation session, participants were asked to listen to and repeat 30 vocoded sentences. Participants then completed practice trials to get accustomed to the task, the speaker’s voice, and the response format. During the practice, participants heard a total of eight sentences, four with accuracy instructions and four with speed instructions. Within each instruction condition, participants heard two sentences with six-channel vocoding and two sentences in clear. None of the practice or adaptation materials were used in the main experiment.

### Results

#### Response accuracy

**Fig. 2:**
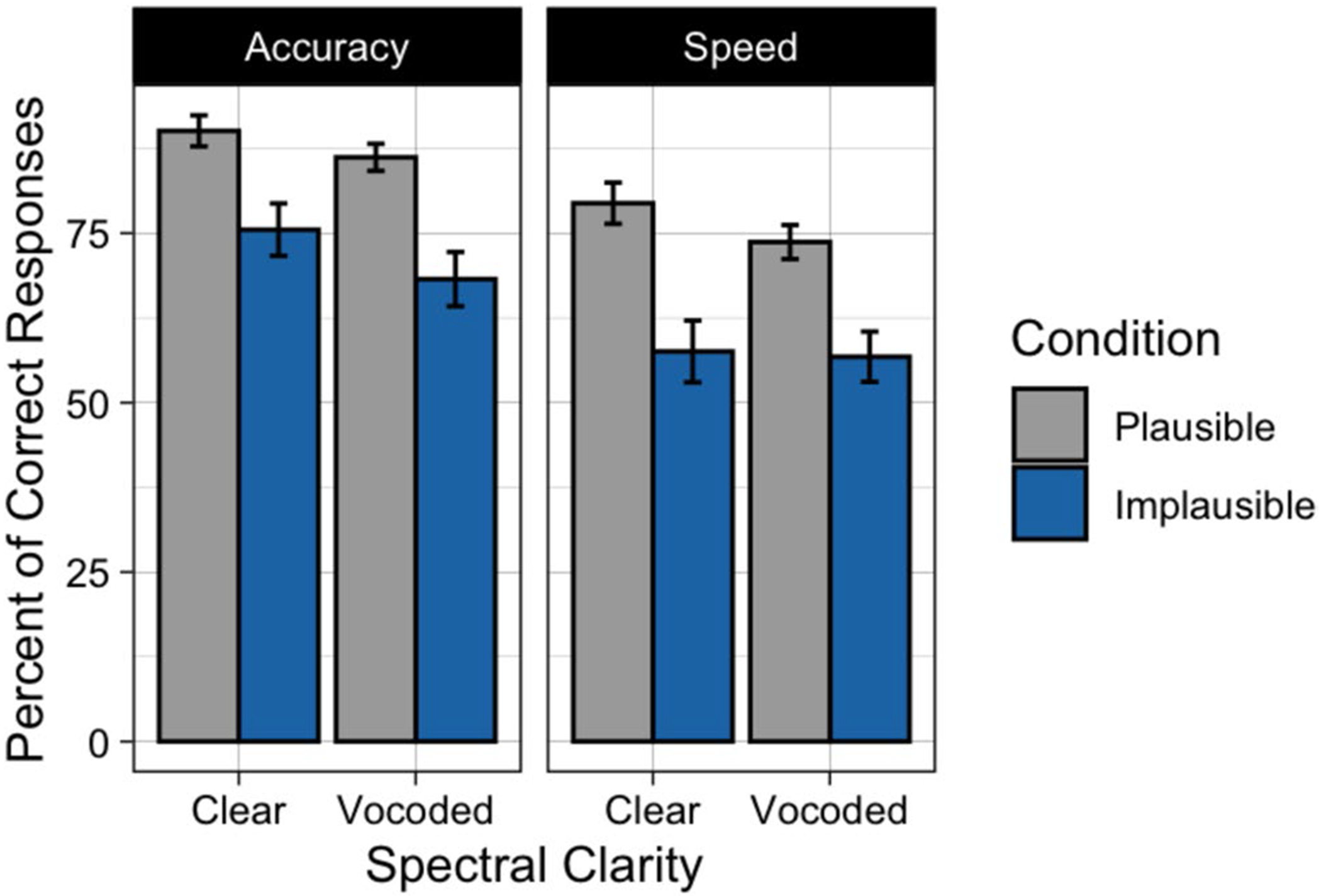
Mean percent of responses when participants chose the literal meaning of the sentence for Experiment 1. Data are shown when speech was heard in clear or vocoded, when participants were given accuracy instructions (**left**) or speed instructions (**right**), and when the sentences were plausible (gray) or implausible (blue). Error bars are one standard error of the mean

Fig. 2 displays the mean percent of responses in which the participant gave the literal interpretation to plausible and implausible sentences when heard with clear or vocoded speech. The left panel shows the data when the orienting instructions stressed accuracy; the right panel when the orienting instructions stressed speed. As expected, visual inspection of Fig. 2 suggests that participants were more likely to give a literal interpretation when the sentences were plausible as compared to when the sentences were implausible. One can also see a tendency for a decrease in literal interpretations when participants were asked to prioritize speed as compared to when they were asked to prioritize accuracy, and a decrease in literal interpretations when the speech was heard with six-channel vocoding as compared to clear speech.

These data were analyzed using generalized mixed effects models, with the binomial family specified using the lme4 package (Bates, 2010). Random effects (intercepts) were included for Participants and Stimuli. A reverse selection approach using likelihood ratio tests was used to select a final most parsimonious model that included only main effects and interactions that were statistically significant. To test the significance of parameters that were included in the final model, null models were created without the specified fixed effect of interest and contrasted with the final model. The final model contained fixed effects of Plausibility (plausible vs. implausible), Clarity (clear vs. vocoded), and orienting Instructions (speed instructions vs. accuracy instructions). The results of the model comparison procedure are shown in Table 1.

Confirming the appearance in Fig. 2, participants’ literal interpretations of sentences significantly decreased when participants were given instructions to prioritize speed relative to accuracy instructions (*p* < 0.001). Giving literal interpretations was more likely when participants listened to sentences with clear speech as compared to sentences that were vocoded (*p* = 0.006). Participants’ responses were more likely to reflect a literal (and syntactically correct) interpretation when the sentences were plausible than when the sentences were implausible (*p* < 0.001). None of the interactions were significant.

#### Response latencies

**Fig. 3:**
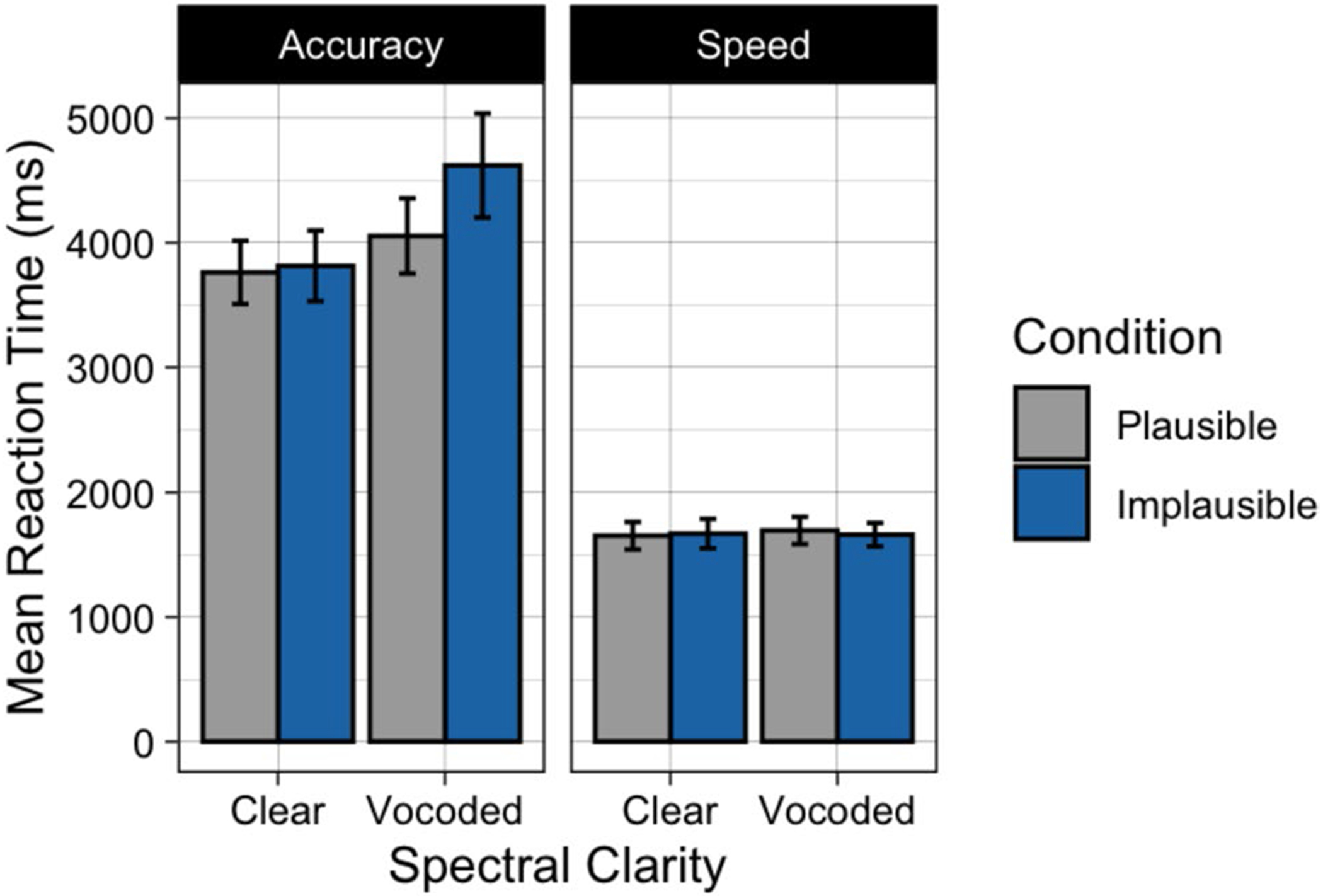
Mean response latency to keypress after sentences were heard in Experiment 1. Data are shown when speech was heard in clear or vocoded, when participants were given accuracy instructions (**left**) or speed instructions (**right**), and when the sentences were plausible (gray) or implausible (blue). Error bars are one standard error of the mean

Participants’ mean latencies to keypress following the true/false question prompt are shown in Fig. 3. Visual inspection suggests that participants were sensitive to the speed instructions, responding over twice as fast when they were asked to prioritize speed compared to when they were asked to prioritize accuracy. When asked to prioritize speed, responses were rapid, with no clear differences in response latencies whether the speech was clear or vocoded, or whether the sentences were plausible or implausible. When asked to prioritize accuracy, there is a hint of a multiplicative effect of plausibility and clarity, resulting in the longest response latencies for the sentences that were both implausible and vocoded.

These data were analyzed using generalized mixed effects models, with the gamma family (log link function) specified using the lme4 package (Bates, 2010). Random effects (intercepts) were included for Participants and Stimuli. The same model comparison procedure was used to apply a reverse selection approach using likelihood ratio tests to select a final most parsimonious model. To test the parameters in the final model, null models were created without the specified fixed effect and contrasted with the final model. The final model for participants’ reaction times contained fixed effects of Clarity (clear vs. vocoded), Instructions (speed instructions vs. accuracy instructions), and the interaction between Clarity and Instructions. The results of the model comparison procedure are shown in Table 2.

There was a nonsignificant trend toward an effect of plausibility (*p* = 0.068), reflecting a tendency for participants to take longer to select their answer when the sentence was implausible than when it was plausible. There was a significant effect of clarity (*p* < 0.001), as evidenced by longer pauses on average when speech was vocoded as compared to clear. Confirming visual inspection, there was a significant effect of instructions (*p* < 0.001), as participants responded significantly faster when asked to prioritize speed and slowed down considerably when asked to prioritize accuracy. There was a significant interaction between task instructions and spectral clarity (*p* = 0.004). This interaction was such that when participants were asked to prioritize speed, pause times were roughly similar between clear and vocoded conditions, but when asked to prioritize accuracy, participants took differentially longer to respond to the vocoded sentences relative to sentences with clear speech. There was a nonsignificant trend for both a plausibility by instructions interaction (*p* = 0.052) and a three-way interaction between plausibility, instructions, and spectral clarity (*p* = 0.073), reflecting the appearance of a differential effect of sentence plausibility on response latency when participants were under accuracy instructions, and when speech was vocoded. The plausibility by clarity interaction was not significant (*p* = 0.222).

#### Drift–diffusion modeling

**Fig. 4:**
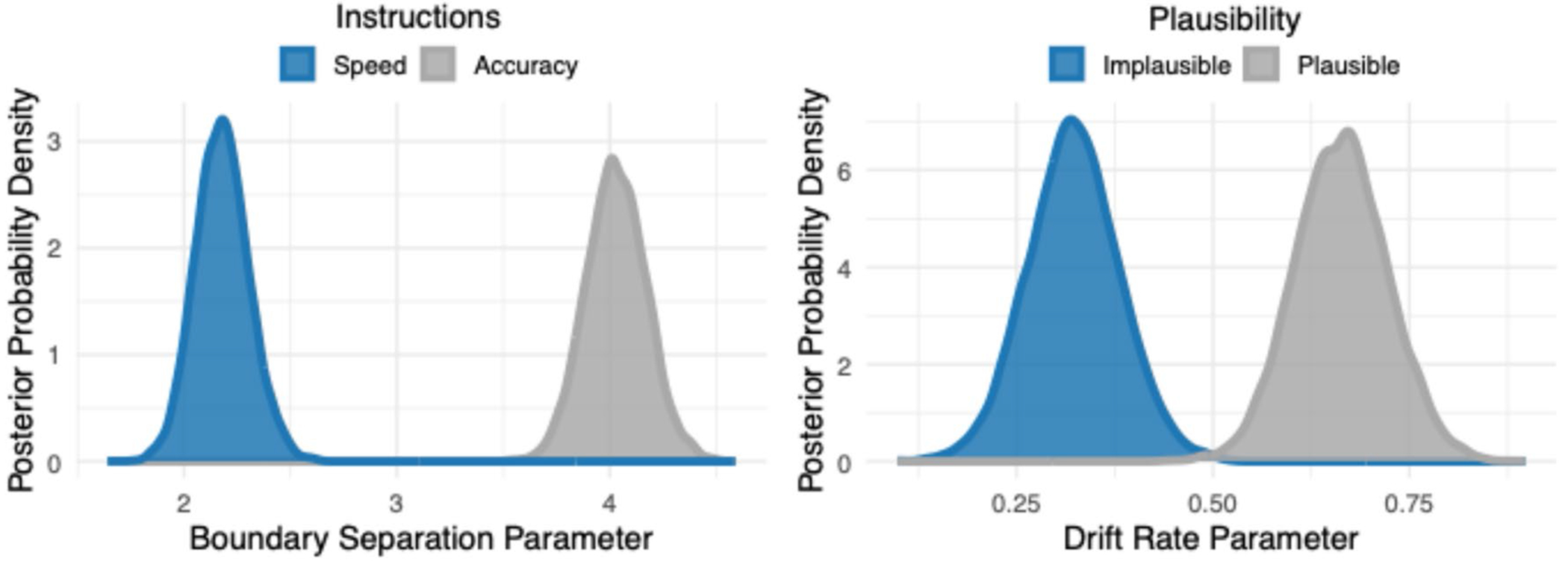
Drift diffusion model results from Experiment 1. Posterior distributions for the boundary separation parameter (**left**) and the drift rate parameter (**right**) from the final hierarchical Bayesian drift diffusion model. Posterior distributions for the boundary separation parameter are shown for when the participants were under instructions to favor speed (blue) and for when participants were asked to favor accuracy (gray). Posterior distributions for the drift rate parameter are shown for when the participants heard sentences that were semantically implausible (blue) and for when sentences were plausible (gray)

As noted, although the behavioral results were intuitively likely, we wished to probe the latent decision-making processes underlying the behavior. For this purpose, we employed drift–diffusion modeling (DDM). Hierarchical Bayesian drift–diffusion models were fit using the HDDM package (Wiecki, et al., 2013) in Python, which uses Monte Carlo Markov Chain (MCMC) simulation to estimate posterior distributions of the effects of our experimental manipulations on the latent decision-making parameters within the DDM framework. We considered a model that allowed for effects of each of the three experimental manipulations (Plausibility, Orienting Instructions, and Speech Clarity) on the boundary separation parameter (a; the threshold at which a decision is made) concurrently with effects of each of the three manipulations on the drift rate parameter (v; the rate of evidence accumulation toward a decision). The initial model was then reduced by removing parameters and model fit was compared using the deviance information criterion (DIC), with the rule to choose the most parsimonious model with the lowest DIC value. Ten thousand simulations were run for each model, with the initial 500 simulations burned. The model comparison procedure is shown in Table 3. The final, most parsimonious drift–diffusion model contained dissociated effects of orienting instructions on boundary separation as well as effects of sentence plausibility on drift rate. The posterior distributions from the final model for the boundary separation and drift rate parameters are shown in Fig. 4.

In the Bayesian approach, statistical significance can be estimated directly from the MCMC traces of the final model by computing the probability that the parameter of interest is greater in each experimental condition (see Kruschke, 2014; Wiecki et al., 2013). To facilitate comparison to the frequentist *p*-value, we used a probability of 0.95 and higher as our indication of statistical significance. As hypothesized, there was a significant effect of orienting instructions on the boundary separation parameter, revealing that participants required more evidence to reach a decision when under accuracy instructions as compared to speed instructions (P_A_[Accuracy > Speed] = 1). There was also a significant effect of sentence plausibility on drift rate, revealing that the amount of evidence accumulation was significantly faster when participants heard plausible sentences as compared to implausible sentences (P_V_[Plausible > Implausible] = 1).

### Discussion

Experiment 1 examined the effects of orienting instructions and spectral clarity on the comprehension of spoken plausible and implausible sentences, with a focus on the role of shallow (“good enough”) processing versus detailed syntactic parsing. We explored these effects by analyzing behavioral accuracy, response latencies, and applying hierarchical Bayesian drift–diffusion modeling to investigate latent decision-making processes. Specifically, we tested the hypotheses that orienting instructions and degraded speech clarity would increase reliance on shallow processing, as reflected in reduced accuracy for implausible sentences. At the same time, we tested the broader effects of these manipulations on parameter shifts in a drift–diffusion framework. We found that both the manipulations of orienting instructions and of speech clarity impacted the likelihood of shallow processing of implausible sentences. This would imply that shallow processing was also being conducted for the plausible sentences. Importantly, drift–diffusion modeling revealed a dissociation where orienting instructions selectively influenced the decision boundary parameter of the model while plausibility selectively influenced the drift rate parameter.

Contrary to our initial hypothesis, we did not find a significant effect of vocoding on the rate of evidence accumulation as indexed by the drift rate parameter. We note that the behavioral effects of spectral degradation were relatively small in this experiment as compared to the effects of task instructions and plausibility. It should be noted that vocoding does not perfectly replicate cochlear implant listening, as further distortions such as perceptual “smearing,” tonotopic (frequency-to-place) mismatch, and neuronal death challenge cochlear implant speech perception (Svirsky et al., 2021).

## Experiment 2

Experiment 1 served as a proof-of-concept demonstration for the impact of spectral clarity and task instructions on the likelihood that an implausible sentence will be erroneously interpreted as having a plausible meaning. Importantly, Experiment 1 also demonstrated that conditions underlying auditory sentence comprehension can be decomposed using a drift diffusion model. Experiment 2 was conducted as a replication of the major findings of Experiment 1, with a focus on the impact of task instructions and sentence plausibility on drift–diffusion model parameters.

### Method

#### Participants

The participants in Experiment 2 were 64 undergraduate students and staff (24 males, 39 females, one non-binary), with ages ranging from 18 to 25 years (*M* = 20.00 years, *SD* = 1.88). Similar to Experiment 1, all participants had a better-ear pure tone threshold average (PTA) across 0.5, 1, 2, and 4 kHz of less than 25 dB HL, placing them in a range considered to be clinically normal hearing for speech (Katz, 2002).

All participants reported themselves to be native speakers of American English with no known medical conditions that might interfere with the ability to perform the experimental tasks. Written informed consent was obtained from all participants according to a protocol approved by the Brandeis University IRB.

#### Stimuli and procedures

The stimuli and procedures used in Experiment 2 were the same as those described in Experiment 1, with one exception. In Experiment 2, the vocoding manipulation was not used. The exclusion of vocoding increased the number of stimuli each participant heard in each condition. For each of the total of 192 sentences a given participant heard, 96 were test sentences. Of the test sentences, 48 were plausible, 24 with accuracy instructions and 24 presented with speed instructions. The other 48 test sentences were implausible, with 24 heard with accuracy instructions and 24 presented with speed instructions. Similar to Experiment 1, the remaining 96 sentences were fillers with 48 presented with accuracy instructions and 48 with speed instructions.

### Results

#### Accuracy

**Fig. 5:**
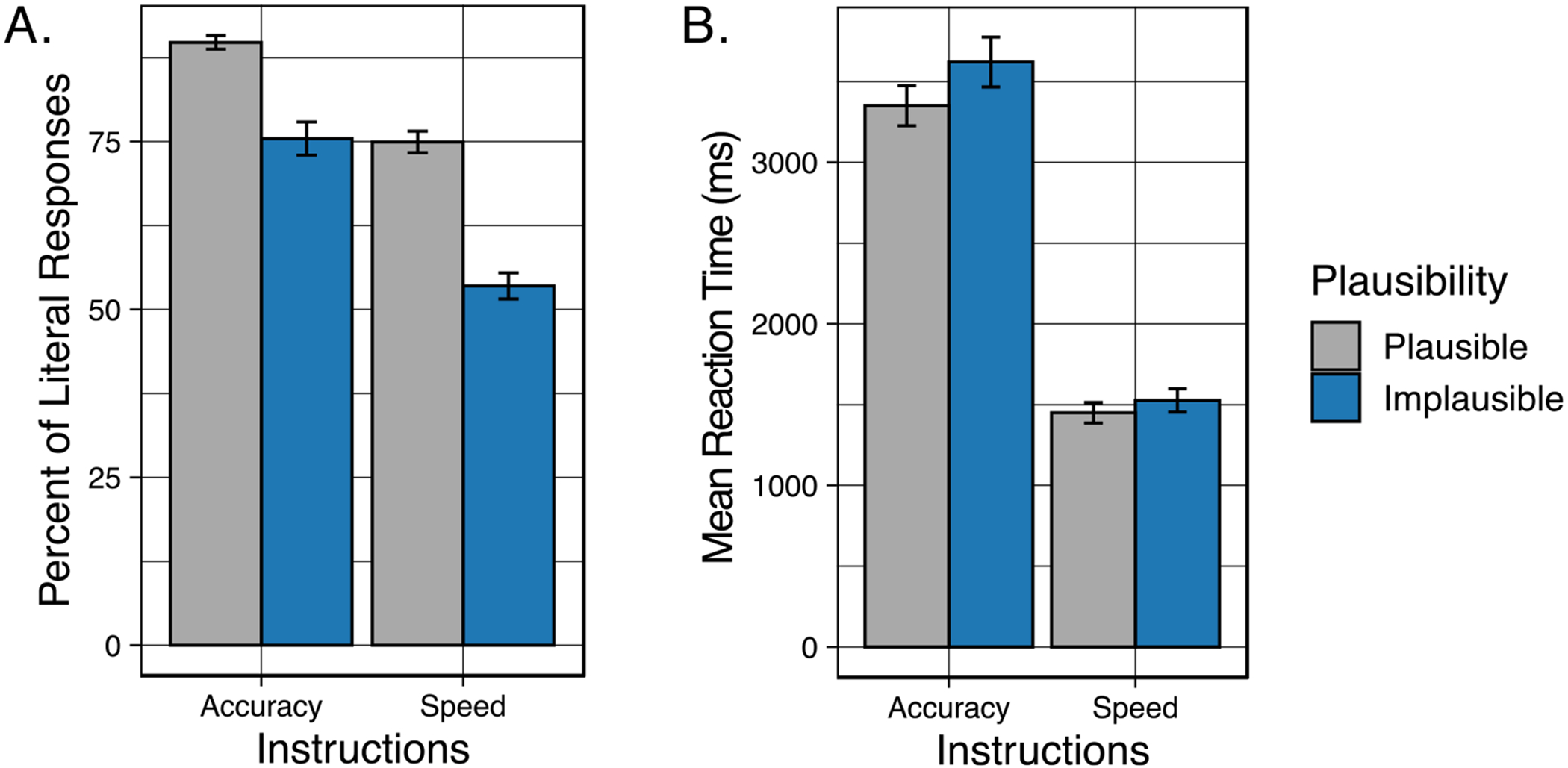
Behavioral results for Experiment 2. (**A**) Mean percent of responses when participants chose the literal meaning of the sentence. Data are shown when speech was when participants were given accuracy instructions or speed instructions, and when the sentences were plausible (gray) or implausible (blue). (**B**) Mean response latency to keypress after sentences were heard. Data are shown when participants were given accuracy instructions or speed instructions, and when the sentences were plausible (gray) or implausible (blue). Error bars are one standard error of the mean

Figure 5(A) displays the mean percent of responses in which the participant gave the literal interpretation to plausible and implausible sentences when heard with instructions that emphasized either the speed or accuracy of the comprehension response. As in Experiment 1, visual inspection of Fig. 5(A) suggests that participants were more likely to give a literal interpretation when the sentences were plausible as compared to when an implausible sentence was heard. The tendency for a decrease in literal interpretations when participants were asked to prioritize speed as compared to when they were asked to prioritize accuracy was also visually replicated.

These data were analyzed using generalized mixed effects models, with the binomial family specified with random effects (intercepts) for Participants and Stimuli. The final model contained fixed effects of Plausibility (plausible vs. implausible) and orienting Instructions (speed instructions vs. accuracy instructions). The results of the model comparison procedure are shown in the first four columns of Table 4. Confirming the appearance in Fig. 5(A), participants’ literal interpretations of implausible sentences significantly decreased when participants were given instructions to prioritize speed relative to instructions that emphasized the accuracy of the response (*p* < 0.001). As demonstrated in Experiment 1, participants’ responses were more likely to reflect a literal interpretation when the sentences were plausible than when the sentences were implausible (*p* < 0.001). The interaction between plausibility and task instructions was not significant (*p* = 0.383).

#### Response latencies

Participants’ mean latencies to keypress following the question prompt are shown in Fig. 5(B). From visual inspection, one can see that when asked to prioritize speed, responses were rapid (around 1,500 ms) with minimal differences in response latencies. When instructions were to prioritize accuracy, response latencies were over twice as long as when they were asked to prioritize speed.

The response latency data shown in Fig. 5(B) were analyzed using generalized mixed effects models, with the gamma family (log link function) specified using the lme4 package (Bates, 2010). Random effects (intercepts) were included for Participants and Stimuli. The final model for participants’ reaction times contained fixed effects of Instructions (speed instructions vs. accuracy instructions), and Plausibility (plausible vs. implausible). The results of the model comparison procedure are shown in Table 4, columns 5–7. The mixed models revealed a significant effect of plausibility (*p* = 0.027), reflecting a tendency for participants to, on average, take longer to select their answer when the sentence was implausible than when the sentence had a plausible meaning. There was a significant effect of instructions (*p* < 0.001), confirming visual inspection that participants responded faster when asked to prioritize speed as compared to when they were instructed to prioritize accuracy. The interaction between task instructions and plausibility was not significant (*p* = 0.336).

#### Drift–diffusion modeling

**Fig. 6:**
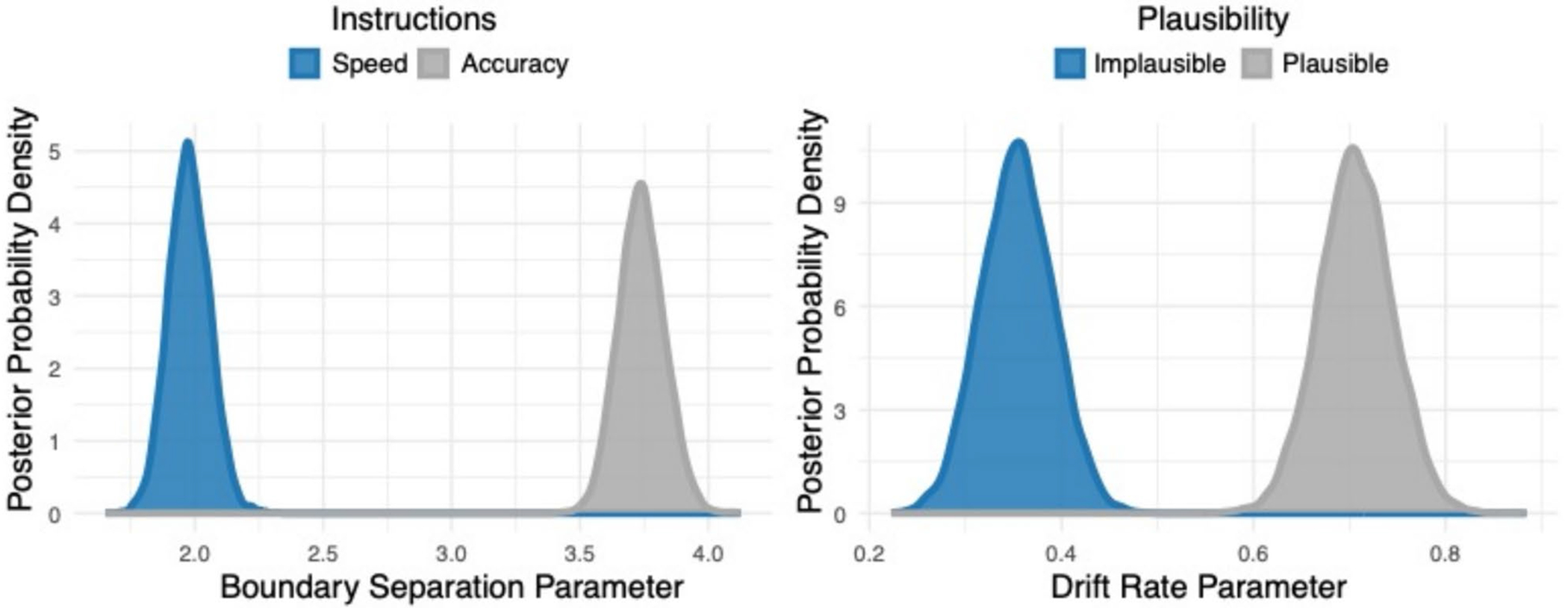
Drift–diffusion results from experiment 2. Posterior distributions for the boundary separation parameter (**left**) are shown for when the participants were under instructions to favor speed (blue) and for when participants were asked to favor accuracy (gray). Posterior distributions for the drift rate parameter (**right**) are shown for when the participants heard sentences that were implausible (blue) and for when sentences were plausible (gray)

To uncover latent decision-making parameters, the accuracy and response latency data were analyzed using drift–diffusion models. The model with dissociable effects of plausibility on drift rate and task instructions on boundary separation was preferred over a full model (DIC difference = 41.52). The posterior distributions of the drift–diffusion model parameters are shown in Fig. 6. Statistical significance was estimated from the MCMC traces from the final model by computing the probability that the parameter of interest was greater in each experimental condition. Using a probability of 0.95 or higher as an index of significance, there was a significant effect of task instructions within the boundary separation parameter with instructions that emphasize accuracy leading to a higher boundary separation than instructions that emphasize speed (P_A_[Accuracy > Speed] = 1). There was also a significant effect of plausibility within the drift rate parameter with plausible sentences leading to a higher drift rate than sentences that were implausible (P_V_[Plausible > Implausible] = 1).

### General discussion

In two experiments, we demonstrated the flexibility of sentence processing and the extent to which conditions that underlie sentence comprehension can be informed using a drift–diffusion modeling account. Our approach was motivated by theories of underspecified, shallow or “good enough” processing, the notion that listeners often rely on plausibility and heuristics rather than a complete syntactic analysis to derive the meaning of a sentence.

The majority of prior work on “good enough” processing has focused on sentences that presented some degree of syntactic challenge (e.g. Amichetti et al., 2016; Ayasse et al., 2021), such that shallow processing may ease participants’ cognitive load. We borrow the term, “principle of least effort” from Zipf (1949), to represent the notion that listeners will often attempt to derive the meaning of an utterance while expending as little effort as conditions allow (Ayasse et al., 2021; see also Brehm & Self, 1989; Richter, 2016). In Experiment 1, we demonstrated that orienting instructions that emphasize rapid responding and perceptual challenge both increase the likelihood that the assumed meaning of a relative clause sentence will be based on plausibility. Regardless of whether orienting instructions emphasized accuracy or speed, participants were more likely to give a literal interpretation to implausible sentences for clear relative to degraded speech. The notable difference was when orienting instructions emphasized speed, there was an overall drop in the proportion of literal interpretations. The effect of orienting instructions on the likelihood that a participants’ comprehension decision would be based on plausibility was replicated in Experiment 2.

A different, albeit not unexpected, picture appeared in the participants’ response latencies. In Experiment 1, we found a general increase in response latencies when sentences were spectrally degraded. In both Experiment 1 and Experiment 2, when participants were instructed to prioritize accuracy, response latencies were significantly longer than when instructed to prioritize speed. With instructions to emphasize speed, responses were rapid, with minimal differences between conditions. This may reflect a minimal time requirement to read the prompt question and execute the response.

The focus of the present study, however, was to determine whether our experimental manipulations influenced the latent decision-making processes underlying the observed accuracy and latency data. The present data demonstrate a unique application of the drift–diffusion model ordinarily associated with decision making tasks (Ratcliff & Rouder, 1998). A critical feature of the drift–diffusion model is the insight that decision making can be characterized as a process of noisily accumulating evidence until a decision threshold has been reached. We argue that this characterization also applies to the listeners’ development of the meaning of an utterance due to the accumulation of evidence from the syntactic and semantic content.

In the present case drift–diffusion modeling revealed a dissociation in which orienting instructions selectively influenced the decision boundary parameter of the diffusion model while plausibility selectively influenced the drift rate parameter. The effects of orienting instructions on the decision boundary and of plausibility on drift rate appear to be robust, as these findings were replicated in the second experiment which was conducted with higher power.

Regarding the decision boundary separation parameter, we found that when instructed to prioritize accuracy, listeners set a higher decision threshold, reflecting a more cautious approach in which the individual accumulated a greater amount of evidence before the decision threshold was reached (e.g., Ratcliff, 2002; Ratcliff & Rouder, 1998; Ratcliff, et al., 2003; Thapar et al., 2003). This finding represents a flexible and strategic adjustment of decision style on the part of the participants to meet the demands of the listening task.

The decision threshold alone was not adequate to fully capture the complexity of the decision-making process in the present experiment, as evidence can be accumulated toward the correct decision boundary within a set threshold at variable rates. In this regard, we observed a selective effect of sentence plausibility on the drift rate parameter, indicating that the rate of evidence accumulation towards the correct decision boundary was significantly faster for plausible as compared to implausible sentences. This is consistent with the previously cited interpretation that shallow processing may ease cognitive load (Ayasse et al., 2021) and reflects the ease of semantic integration in plausible sentences relative to implausible sentences. Importantly, the dissociation between drift rate and boundary separation suggests that the change in drift rate due to plausibility is not a strategic decision to respond rapidly on the part of the participants, but a shift in the speed of integration due to the quality or coherence of the linguistic content of the speech signal.

There is an open debate as to whether so-called “thematic role reversal errors” occur during the initial parse of the sentence or arise post-interpretively from memory when the participants are presented with the comprehension probe (Meng & Bader, 2021). Recent eye-tracking results from Christianson and colleagues (2023), which manipulated role reversal during online reading, favor the parsing account, suggesting that thematic role reversal errors reflect early misinterpretations in the initial construction of the sentence meaning. The present results are suggestive of this interpretation, albeit not conclusive. That is, if the issue was primarily memory retrieval at the probe stage, one might expect participants to compensate for this difficulty by taking extra time to accumulate more evidence before committing to a response. In the present case, no corresponding increase in boundary separation was evident for the implausible sentences, suggesting that there was no strategic or meta-cognitive adjustment. However, it is important to note that the drift–diffusion model used in the present study was not sensitive to the precise moment within the trial when the thematic role reversal error may have occurred, and thus cannot definitively answer this question. Future research in this area may consider combining drift–diffusion models with a time-sensitive measure such as eye tracking, self-paced reading, or event-related potentials to cohesively map the temporal dynamics of ‘good enough’ processing onto decision making components.

It is important to note that the present experiment contained probe statements following the auditorily presented sentence that were counterbalanced such that the probe statements were equally likely to be plausible or implausible. This design choice was made to prevent a predictable pattern in the probe statements that could lead participants to rely solely on the probe rather than the spoken sentence. As a result of this counterbalancing, half of the plausible trials included implausible probes, and half of the implausible trials included plausible probes. However, because the probes were distributed evenly across conditions, we do not expect a systematic effect on the results. Additionally, the probes were intentionally designed to be simpler than the experimental sentences. They were short, syntactically straightforward, and remained visible on screen until a response was made. In contrast, the spoken sentences were longer, more complex, and transient. As such, we maintain that the thematic role reversal errors observed in this experiment are more likely attributable to the processing demands associated with the spoken sentences rather than the probes themselves.

Taken together, our results demonstrate that sentence processing algorithms are flexible, such that under certain conditions a syntactic representation of a sentence precedes comprehension and under other conditions a process of sampling, inference, and plausibility is used. At the theoretical and methodological level, these results show that one can decompose factors underlying complex listening behavior using a drift–diffusion model. To our knowledge, this is the first use of drift–diffusion modeling to understand the effects of plausibility in auditory sentence comprehension.

## Figures and Tables

**Table 1:** Log-likelihood model comparisons for generalized linear mixed effects analysis of accuracy performance in Experiment 1

Predictor	*χ* ^2a^	*df* ^b^	*p* ^c^
Plausibility	105.78	1	**< 0.001**
Clarity	7.53	1	**0.006**
Instructions	82.44	1	**< 0.001**
Plausibility X Clarity	1.79	1	0.181
Plausibility X Instructions	0.69	1	0.407
Clarity X Instructions	1.85	1	0.173
Plausibility X Clarity X Instructions	0.78	1	0.378

a χ^2^ value for comparisons of each step of the model

b Degrees of freedom for the χ^2^ test

c *p*-Value reflects significance of change in model fit at each step of the model. Significant *p*-values of are indicated in bold

**Table 2:** Log-likelihood model comparisons for generalized linear mixed effects analysis of response latencies for Experiment 1

Predictor	*χ* ^2a^	*df* ^b^	*p* ^c^
Plausibility	3.33	1	0.068
Clarity	27.13	2	**< 0.001**
Instructions	1934.2	2	**< 0.001**
Plausibility X Clarity	1.49	1	0.222
Plausibility X Instructions	3.76	1	0.052
Clarity X Instructions	8.16	1	**0.004**
Plausibility X Clarity X Instructions	2.20	1	0.073

a χ^2^ value for comparisons of each step of the model

b Degrees of freedom for the χ^2^ test

c *p*-Value reflects significance of change in model fit at each step of the model. Significant *p*-values of are indicated in bold

**Table 3:** Model comparison procedure for the hierarchical Bayesian drift diffusion models for Experiment 1

Model description	*D.I.C*	Model selected
Model 1. Full Model	11691.88	Model 1
Model 2. Vocoding removed completely	11653.76	Model 2
**Model 3. Instructions removed from drift rate, plausibility removed from decision boundary**	**11628.28**	**Model 3**
Model 4. Plausibility removed completely	11782.45	Model 3
Model 5. Instructions removed completely	13154.86	Model 3

*D.I.C.* = Deviance Information Criterion

Final model shown in bold

**Table 4:** Log-likelihood model comparisons for generalized linear mixed effects analysis of behavioral performance for Experiment 2

Predictor	Accuracy			Reaction time		
	*χ* ^2a^	*df* ^b^	*p* ^c^	*χ* ^2a^	*df* ^b^	*p* ^c^
Plausibility	316.98	1	**< 0.001**	4.90	1	**0.027**
Instructions	81.43	1	**< 0.001**	3906.40	1	**< 0.001**
Plausibility X Instructions	0.76	1	0.383	0.82	1	0.366

a χ^2^ value for comparisons of each step of the models

b Degrees of freedom for the χ^2^ test

c *p*-Value reflects significance of change in model fit at each step of the model. Significant *p*-values of are indicated in bold

## Data Availability

The data and materials for the experiment are available to other researchers upon request.

## Code availability

The software and custom code supporting these conclusions in this manuscript will be available to other researchers upon request.
